# Clinical and radiologic outcomes of the modified phemister procedure with coracoclavicular ligament augmentation using mersilene tape versus hook plate fixation for acute acromioclavicular joint dislocation

**DOI:** 10.1186/s12893-022-01808-4

**Published:** 2022-10-29

**Authors:** Yu-Jui Chang, Wen-Yi Chou, Jih-Yang Ko, Hao-Chen Liu, Ya-Ju Yang, Ka-Kit Siu

**Affiliations:** 1 Department of Orthopaedic Surgery, Kaohsiung Chang Gung Memorial Hospital, Chang Gung University College of Medicine, Kaohsiung, Taiwan; 2 Department of Medical Research, Kaohsiung Chang Gung Memorial Hospital, Chang Gung University College of Medicine, Kaohsiung, Taiwan; 3 Department of Orthopaedic Surgery, Park One International Hospital, Kaohsiung, Taiwan

**Keywords:** Acromioclavicular joint dislocation, Hook plate, Modified Phemister procedure

## Abstract

**Background:**

The clinical superiority of surgical treatment for acromioclavicular (AC) joint dislocation remains controversial. The aim of this study was to compare the clinical and radiological outcomes of the modified Phemister procedure with CC ligament augmentation using Mersilene tape to those of hook plate fixation for acute AC joint dislocation.

**Methods:**

In this study, patients who received modified Phemister surgery with CC ligament augmentation using Mersilene tape (PM group) or hook plate fixation (HK group) for acute unstable AC joint dislocation with a minimum 5-year follow-up period were retrospectively reviewed. The clinical outcomes were evaluated according to blood loss during surgery, surgical duration, visual analogue scale (VAS), Constant-Murley score (CMS), University of California at Los Angeles (UCLA) shoulder rating scale, and the occurrence of complications. Radiological outcomes were assessed from radiographs according to multiple parameters, including CC distance maintenance, acromion osteolysis, and the presence of distal clavicle osteolysis.

**Results:**

A total of 35 patients completed follow-up for more than 5 years and were analyzed in this study (mean = 74.08 months). There were 18 patients in the PM group and 17 in the HK group. The PM group exhibited similar improvement in functional outcome to the HK group. Regarding radiological outcomes, the HK group had a superior performance in terms of CC distance maintenance, of statistical significance (CCDR: 94.29 ± 7.01% versus 111.00 ± 7.69%, *p* < 0.001) after a one-year follow-up period. However, there were 4 cases of acromion osteolysis and 2 cases of distal clavicle osteolysis in the HK group.

**Conclusion:**

Hook plate fixation was found to be superior to the modified Phemister technique with CC ligament augmentation using Mersilene tape in terms of CC distance maintenance, but there was no significant difference in the functional outcome after 5 years of follow-up. Both surgical methods are reliable options for the treatment of acute AC joint dislocation. Modified Phemister surgery with CC ligament augmentation using Mersilene tape is a relatively lower-cost option for acute AC joint dislocation without the need of a second surgery for implant removal.

## Background

Acromioclavicular (AC) joint dislocation is a common injury, accounting for approximately 10% of all shoulder girdle injuries in clinical practice [1]. It occurs more frequently in young, active populations. Treatment of AC joint dislocation is usually according to the Rockwood classification system [2] and the functional demand of the patient [3]. Surgical interventions for advanced AC joint injuries are divided into vertical coracoclavicular (CC) reconstruction, horizontal AC reconstruction, or a combination of both axes [4]; however, there is no evidence of absolute superiority of one technique over another [3, 5]. Common procedures for vertical CC reconstruction include CC ligament repair, Bosworth screw fixation, and CC ligament augmentation or reconstruction using artificial materials, autograft or allograft, etc. For horizontal AC joint reconstruction, trans-acromial fixation is the major principle, which is accomplished using several modalities, such as Knowles pinning, Kirschner wire pinning using the tension band technique, and hook plate fixation [6, 7]. Otherwise, arthroscopic-assisted surgery presents an alternative intervention and also has good results [8–10].

The Phemister [11] or modified Phemister technique [12–14] has gained in popularity due to its low cost, simple modality, and satisfactory clinical outcome for the treatment of acute AC joint dislocation. CC ligament augmentation [3, 15, 16] with a suture anchor [13] or Mersilene tape [17] also provides effective clinical results. However, soft tissue damage and related blood loss, CC ligament calcification, and clavicle osteolysis [17] have been reported; in addition, involvement of the coracoid process may lead to axillary nerve compromise [18]. The hook plate, which features a trans-subacromial hook engagement under the bottom of the acromion, is commonly used nowadays due to its advantages of providing high stability of the AC joint and good functional recovery [19, 20]. However, complications such as subacromial impingement and rotator cuff damage [21], acromion osteolysis and further acromion fracture [22, 23], and clavicle fracture following a retained implant [24] have been reported. The need for a second surgery for implant removal is also a concern for patients and physicians.

The (modified) Phemister technique, CC ligament augmentation, and hook plate fixation are common procedures with good clinical outcomes. Although there exists comparative research regarding these common surgical modalities in terms of postoperative outcome and complications [25, 26], the treatment options remain under debate and the mid-term clinical effectiveness still need to be addressed. No study has been performed to compare the outcome of the modified Phemister procedure with CC ligament augmentation using Mersilene tape with hook plate fixation. Otherwise, these two procedures are the most common used surgical method in our institution currently. Therefore, we decide to evaluate these two procedures in this series. We hypothesized that the modified Phemister procedure with CC augmentation using Mersilene tape provides similar clinical and radiological outcomes to hook plate fixation in the treatment of acute AC joint dislocation.

## Materials and methods

### Patient enrollment and data collection

**Fig. 1:**
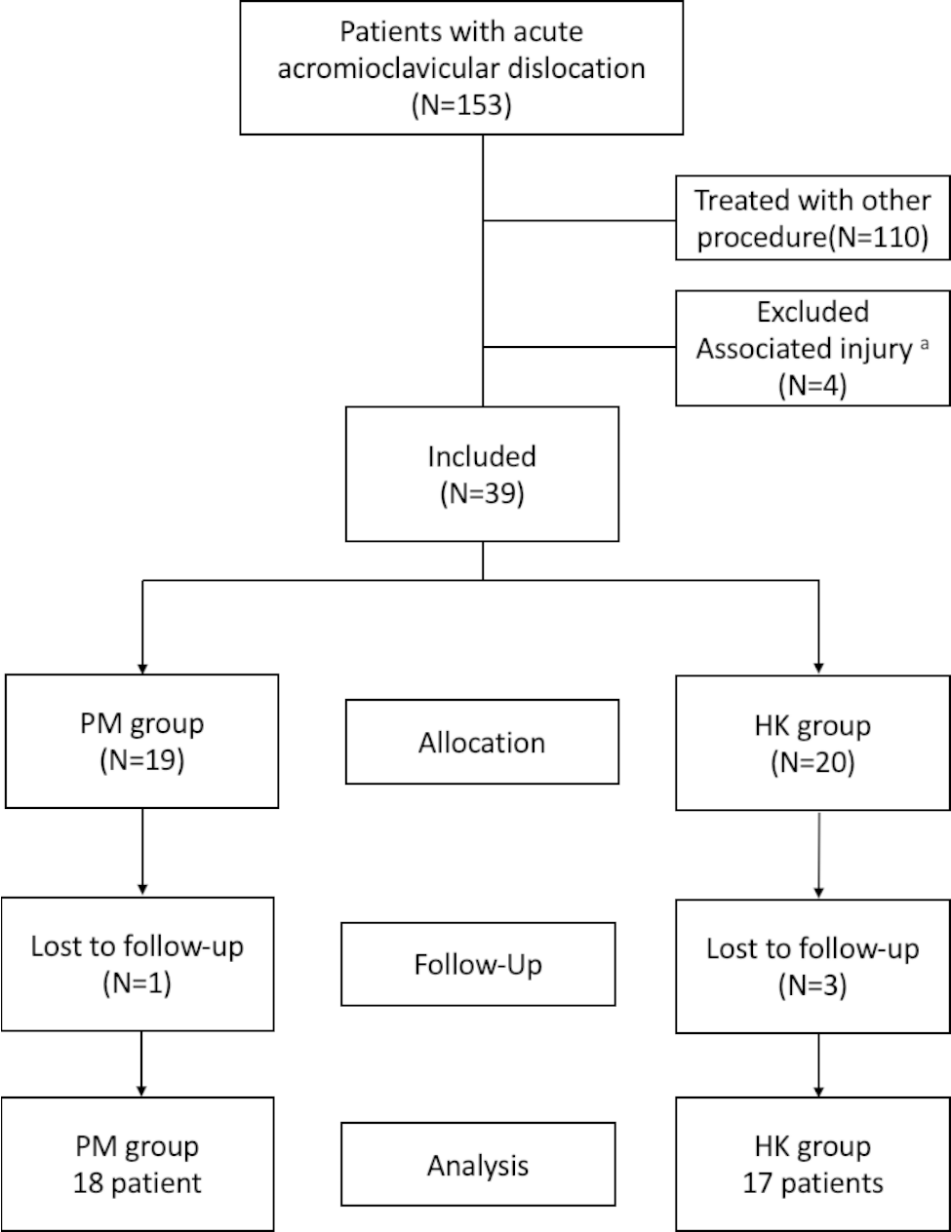
Flowchart of the enrollment of patients. ^a^There were two patients diagnosed with ipsilateral scapula fracture, one patient with acromion fracture, and one patient with proximal humerus fracture excluded

Institutional review board approval was obtained prior to initiation of the study. This retrospective study was conducted at a single, urban, level 1 trauma center. From January 2010 to December 2016, patients with acute (≤ 4 weeks) AC dislocation (type III–VI) undergoing surgical intervention were enrolled for analysis. Those who were treated with percutaneous trans-acromion Kirschner wires and CC ligament augmentation with synthetic Mersilene tape (Ethicon, Somerville, NJ, USA) were allocated to the PM group, while patients treated with hook plate fixation (DePuy Synthes 3.5-mm Clavicle Hook Plate or Aplus® Distal Clavicle HOOK Locking Plate System) were allocated to the HK group. Patients with the following conditions were excluded: (1) previous shoulder surgery, (2) preoperative shoulder stiffness, (3) glenohumeral joint arthritis, (4) associated fracture of the ipsilateral clavicle, scapula, coracoid process, acromion, or proximal humerus, or (5) fewer than 5 years of follow-up. Patient demographic data, including age, gender, injury mechanism, and type of AC dislocation according to the Rockwood classification [2] were recorded. Finally, a total of 153 patients who sustained acute unstable acromioclavicular dislocation and underwent surgical intervention were enrolled. 110 patients who were operated upon using other surgical procedures such as the modified Bosworth procedure were excluded. Four patients were excluded due to associated injuries, including ipsilateral scapula (N = 2), acromion (N = 1), or proximal humerus (N = 1) fracture. A total of 39 patients were enrolled; 19 were allocated into the PM group and 20 into the HK group. After 5 years of follow-up, there were 18 patients in the modified Phemister technique with CC augmentation group (lost to follow-up, N = 1) and 17 patients in the hook plate fixation group (lost to follow-up, N = 3) for the final analysis (Fig. 1).

### Surgical technique

**Fig. 2:**
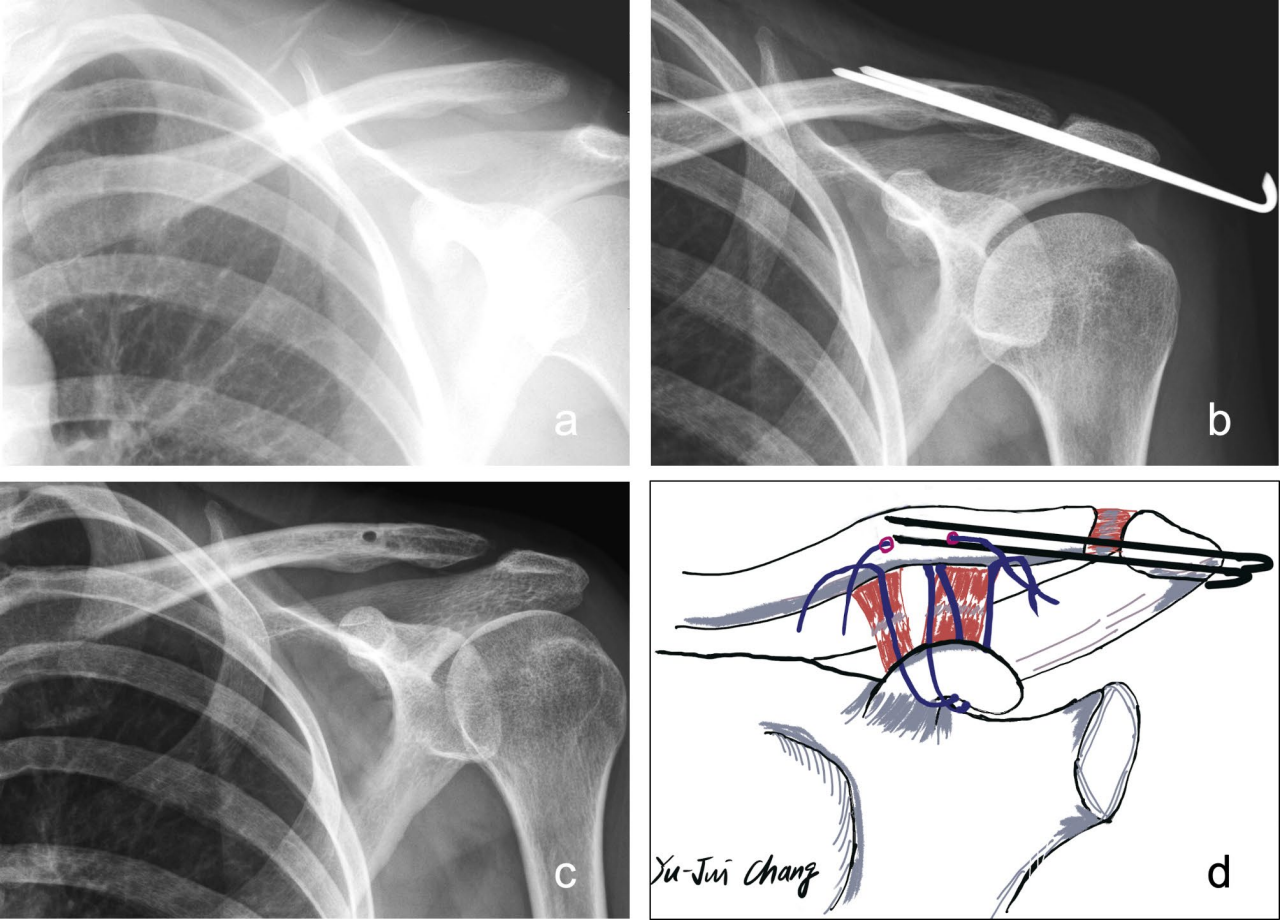
The modified Phemister operation and CC ligament augmentation with Mersilene tape for a type III AC joint dislocation. **(a)** Preoperative plain radiograph. **(b)** Postoperative 1-month plain radiograph. **(c)** Postoperative 1-year plain radiograph. **(d)** The surgical illustration we adopted in this study

Under general anesthesia, all patients were placed in a beach-chair position on an ordinary operating table. The approach began with a transverse incision along the acromion to the coracoid process. In the modified Phemister technique with CC ligament augmentation group (PM group), two percutaneous trans-acromial 1.8-mm or 2.0-mm smooth Kirschner wires were first applied to achieve reduction. Then, two Mersilene tape sutures were passed from the undersurface of the coracoid using a curved passer after splitting the deltoid to the two bone tunnels of the clavicle created using a 3.5-mm drill-bit. Fixation of the Mersilene tapes on the distal clavicle was performed on the remnants of the CC ligaments with node-tying on the anteroinferior side of the distal clavicle after anatomic reduction of the AC joint (Fig. 2). The Kirschner wires were removed 6 to 8 weeks postoperatively at an out-patient clinic without the need for secondary surgery and anesthesia.

**Fig. 3:**
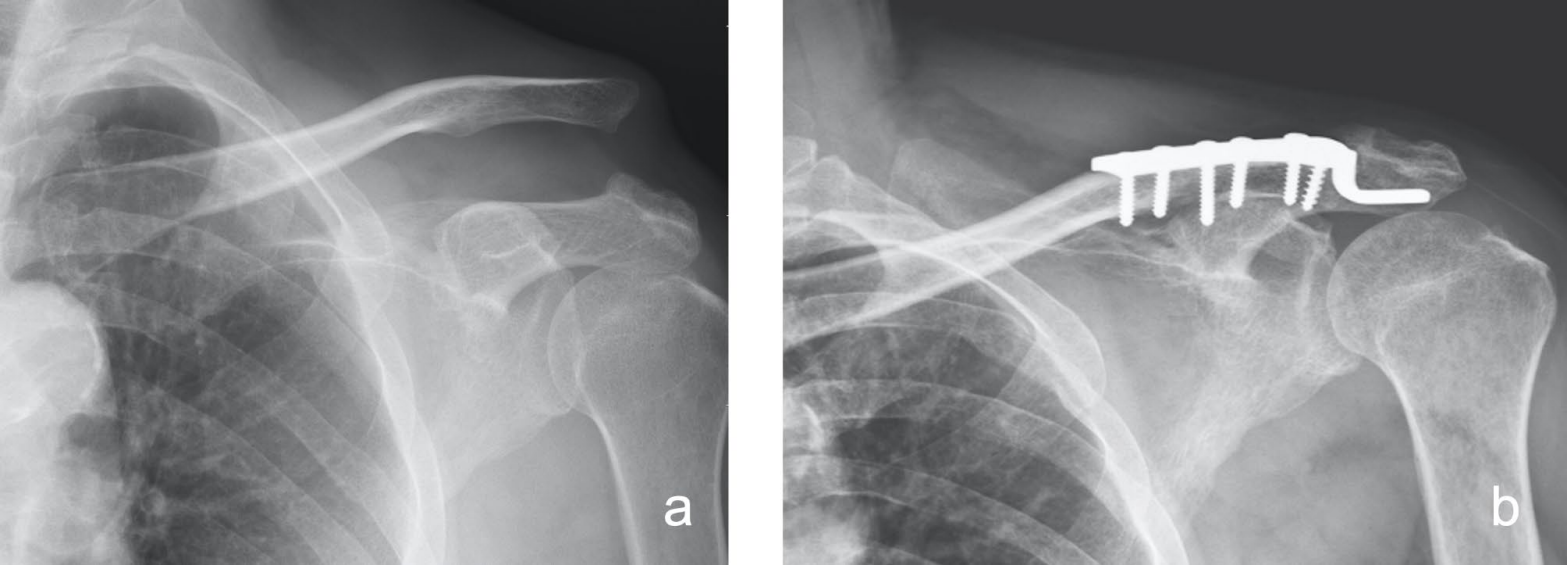
The hook plate fixation modality for a type V AC joint dislocation. **(a)** Preoperative plain radiograph. **(b)** Postoperative 1-month plain radiograph

In the hook plate fixation group (HK group), the hook part of the plate was inserted on the posteroinferior site of the acromion after reduction of the AC joint, and fixation screws were then applied for definite fixation (Fig. 3). After the implant had been inserted, the wound was closed layer by layer, including repair of the torn delto-trapezial fascia in both groups. The implanted hook plates were scheduled to be removed 4 to 6 months after the index surgery.

### Aftercare and rehabilitation

After the surgery, analgesic therapy was administered to patients as required, and a shoulder sling was employed for 6 weeks in every case. Passive range of motion exercise was initiated after the day of the surgery, and passive-assisted motion started from the third week after surgery. Active motion of greater than 90 degrees could be carried out 4 weeks after the surgery as tolerated by the patient.

### Clinical and radiographic evaluations

**Fig. 4:**
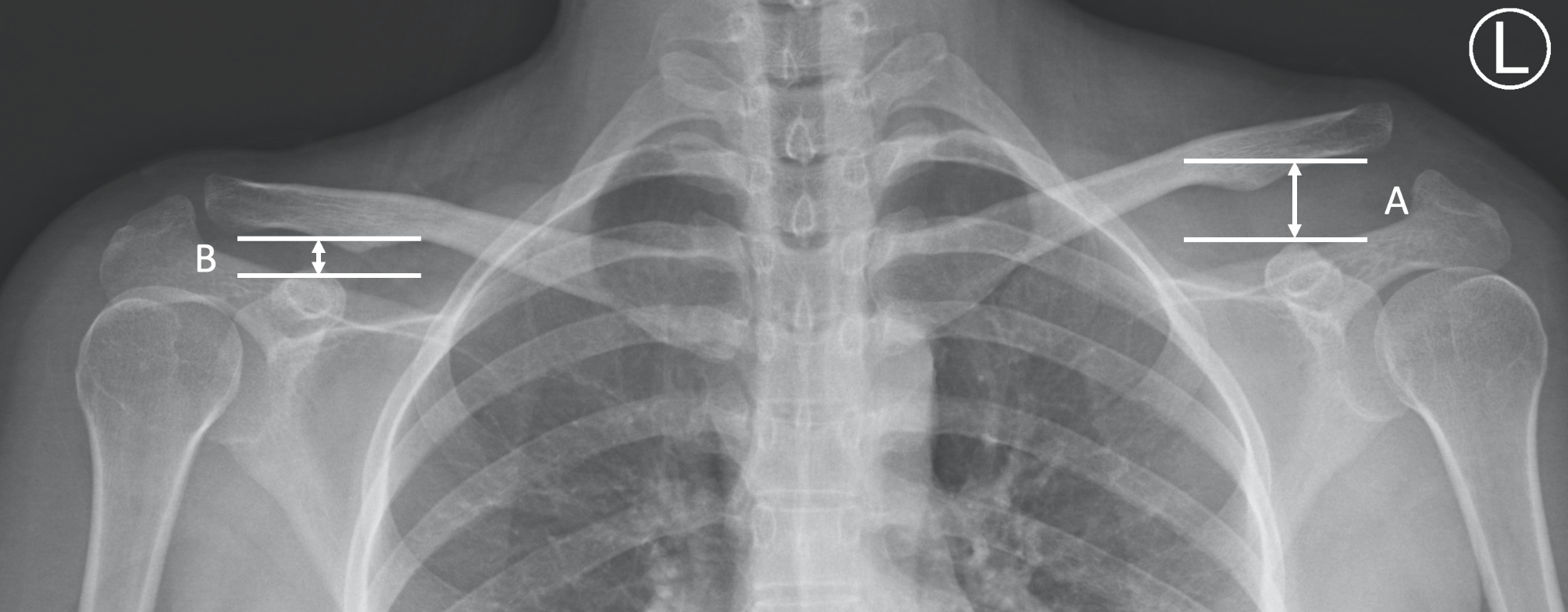
The coracoclavicular distance discrepancy ratio (CCDR= (A/B) x 100%). CCDR is the ratio of coracoclavicular distance of injured shoulder(A) over contralateral healthy side(B)

The surgical duration, intraoperative blood loss, and occurrence of complications were documented for surgical parameter comparison. Functional outcome assessment included analysis of the VAS, Constant-Murley score [27] and UCLA score [28], which were obtained at least 5 years postoperatively. Radiological outcome evaluation was based on the CCDR (Fig. 4) measured preoperatively and at 1, 3, 6 and 12 months after surgery on X-ray.

The coracoclavicular (CC) distance is measured from the uppermost border of the coracoid process to the opposing clavicle surface. To minimize individual difference, the ratio of the injured shoulder CC distance over the normal contralateral shoulder CC distance, which is described as the CCDR [29], was adopted for accurate analysis (Fig. 4). Complications such as infection or implant failure and postoperative radiographic changes, including acromial or distal clavicle osteolysis, and residual subluxation (CCDR > 100%) [17, 26] were documented. All measurements and interpretations of radiographs were repeated twice independently and averaged by two orthopedic trauma research fellows who did not participate in any surgical procedure.

### Statistical analysis

In this study, statistical analysis was completed using the software (version 20.027; MedCalc, Ostend, Belgium). Data were analyzed using the Shapiro-Wilk test to assess the normality of the distribution. Continuous variables are presented as the mean and standard deviation (SD). The Fisher exact test or the chi-square test was used to compare categorical variables. The Wilcoxon signed-rank test or the paired Student *t* test was used to compare differences before and after surgery. The Mann-Whitney *U* test or independent Student *t* test was used to compare outcomes between the two groups. Null hypotheses were rejected when *p*-values were < 0.05.

## Results

### Patient demographics and clinical characteristics

A total of 153 patients who sustained acute AC joint dislocation and underwent surgical intervention were included in this study, 18 patients in the PM group, and 17 patients in the HK group (Fig. 1). The average age of the patients was 43.54 years at the time of surgery. The duration from injury to surgery was 8.33 ± 5.31 days in the PM group and 2.71 ± 3.72 days in the HK group, the difference being significant (*p* = 0.010). The average follow-up period was 75.95 ± 12.99 months in the PM group and 72.10 ± 12.79 in the HK group, which indicated no significant difference between groups (*p* = 0.2157). The majority of injuries were sustained in traffic accidents. The distribution of injury type was as follows: 14 patients of type III and 21 of type V (Table 1).

**Table 1:** Patient demographic data

	PM group	HK group	***p-***value
Number of patients	18	17	
Age	45.33 ± 11.36	41.65 ± 14.87	0.4142
Gender			0.2889
Males	10	13	
Females	8	4	
Site			0.7332
Left	12	10	
Right	6	7	
Injury Mechanism			0.6581
Fall-down	2	3	
Traffic accident	16	14	
Rockwood classification			0.7332
III	8	6	
V	10	11	
Time to surgery (days)	8.33 ± 5.31	2.71 ± 3.72	0.0010
Follow up (months)	75.95 ± 12.99	72.10 ± 12.79	0.2157

### Clinical outcomes

The HK group demonstrated a significantly shorter surgical duration and less blood loss as compared with the PM group (surgical duration: 98.00 ± 27.32 versus 125.72 ± 25.61 min, *p* = 0.0040; blood loss: 15.88 ± 15.73 versus 44.89 ± 27.91 ml, *p* = 0.0003) (Table 2). Both groups revealed a similar VAS (0.5556 ± 0.7048 vs. 0.9412 ± 0.8269, *p* = 0.1538) and functional outcomes, including the Constant-Murley score (95.83 ± 4.03 versus 96.29 ± 3.10, *p* = 0.9602) and UCLA score (32.39 ± 2.45 versus 31.82 ± 1.81, *p* = 0.1595), at the final follow-up.

**Table 2:** Clinical outcomes

	PM group	HK group	***p***-value
Surgical parameter			
Surgical time(min)	125.72 ± 25.61	98.00 ± 27.32	0.0040
Blood loss(ml)	44.89 ± 27.91	15.88 ± 15.73	0.0003
Functional parameter			
VAS	0.5556 ± 0.7048	0.9412 ± 0.8269	0.1538
UCLA Score	32.39 ± 2.45	31.82 ± 1.81	0.1595
Constant-Murley Score	95.83 ± 4.03	96.29 ± 3.10	0.9602
Complications			
Infection	none	none	-
Implant failure	none	none	-
Residual subluxation	15	3	0.0002
Acromion osteolysis	0	4	0.0454
Distal clavicle osteolysis	0	2	0.2286

### Radiological outcomes

**Fig. 5:**
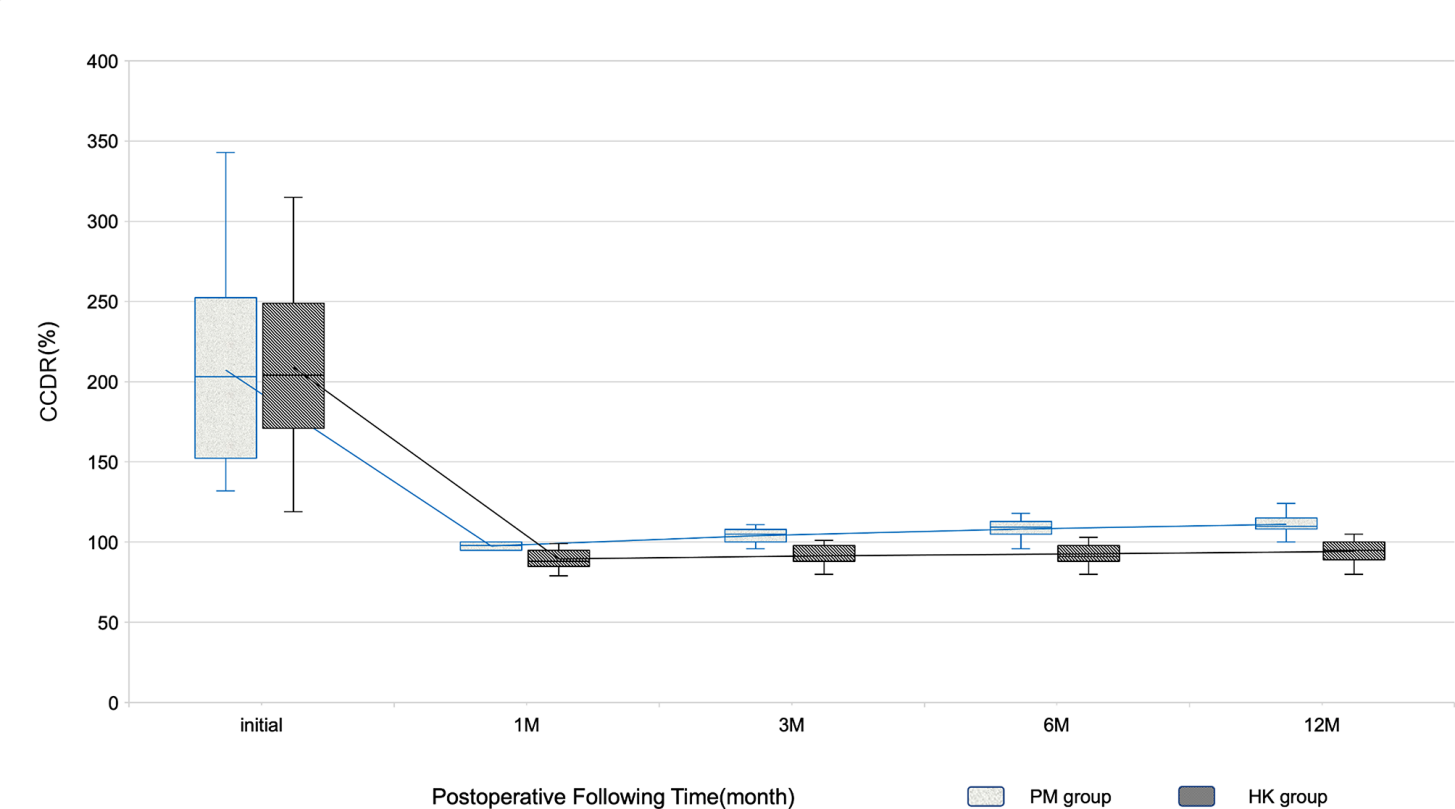
The line chart showed the serial change of CCDR in both groups before surgery and by postoperative follow-up time

**Fig. 6:**
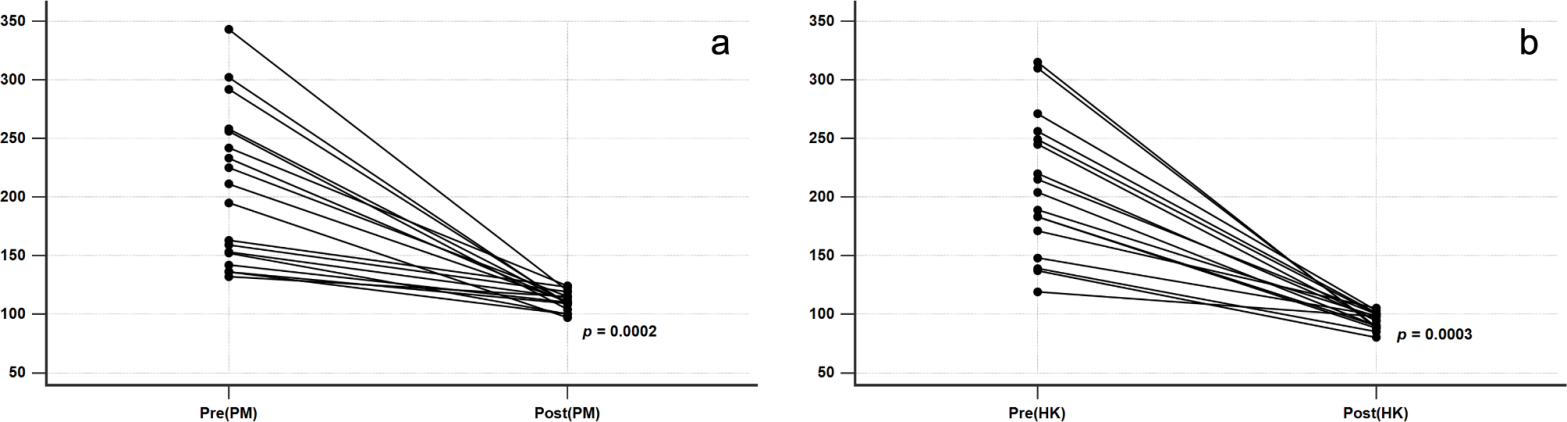
Correction of CCDR before surgery and postoperative 12 months in both groups. Both groups showed significant improvement of CCDR.

**Fig. 7:**
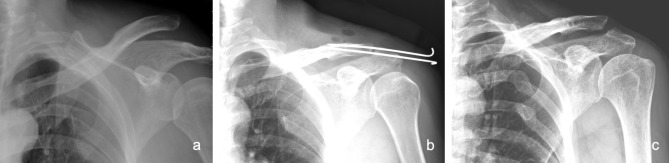
A 44 years old male presented with left shoulder residual subluxation after 4 months of K-wires removal. **(a)** Preoperative plain radiograph. **(b)** Status post open reduction and fixation with K-wires and CC ligament augmentation with Mersilene tape. **(c)** Residual subluxation after 4 months of K-wires removal. However, there was no clinical instability of the patient shoulder

Regarding radiographic analysis, there was no difference in the CCDR between the two groups before surgery (PM group to HK group: 207.22 ± 65.19 to 209.06 ± 58.99, *p* = 0.9310) (Table 3). The group HK presented a significantly superior reduction maintenance at each postoperative follow-up point (Fig. 5) (Table 3). However, both groups showed significant improvement in the CCDR after 12 months of the index surgery (*p* < 0.05 in both groups) (Fig. 6). Meanwhile, the number of patients with residual subluxation was higher in the PM group (Fig. 7), and the difference was statistically significant (*p* = 0.0002) (Table 2).

**Table 3:** Radiological outcomes

CCDR (%)	PM group	HK group	*p*-value
Preop	207.22 ± 65.19	209.06 ± 58.99	0.9310
Postop 1 month	97.56 ± 2.33	89.53 ± 6.40	< 0.0001
Postop 3 months	104.6 ± 4.98	91.47 ± 6.19	< 0.0001
Postop 6 months	108.17 ± 6.47	92.76 ± 6.63	< 0.0001
Postop 12 months	111.00 ± 7.69	94.29 ± 7.01	< 0.0001
∆CCDR^a^	96.22 ± 64.50	114.76 ± 58.57	0.3806

^a^∆CCDR means the correction between preop and postop 12 months CCDR.

**Fig. 8:**
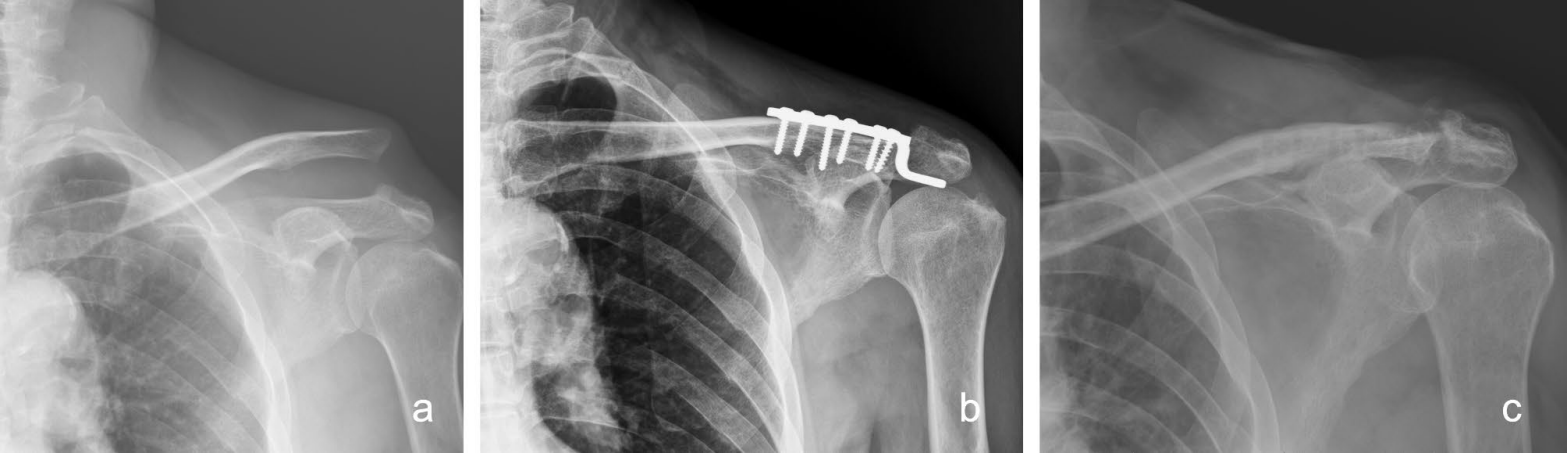
A 62 years old male presented with left shoulder acromion osteolysis after hook plate fixation. **(a)** Preoperative plain radiograph. **(b)** Status post open reduction and fixation with hook plate. **(c)** Acromion osteolysis noted at postoperative 1-year follow up

**Fig. 9:**
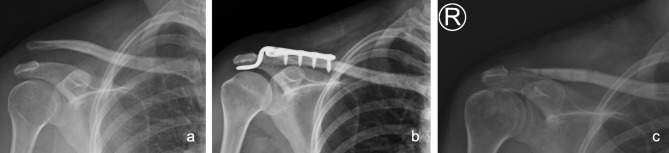
A 49 years old female presented with right shoulder distal clavicle osteolysis after hook plate fixation. **(a)** Preoperative radiography. **(b)** Status post open reduction and fixation with hook plate. **(c)** Distal clavicle osteolysis noted at postoperative 1-year follow up

**Complications** Among the patients who underwent surgery via the modified Phemister technique with CC ligament augmentation, no acute infections or other complications such as implant failure occurred. With regards to the hook plate group, 4 patients sustained acromial osteolysis (Figs. 8) and 2 developed distal clavicle osteolysis (Fig. 9). The results implied that hook plate fixation offered a superior outcome in terms of reduction maintenance but with more frequent acromial complications.

## Discussion

To the best of our knowledge, this was the first study in which the clinical and radiological outcomes of the modified Phemister procedure with CC ligament augmentation using Mersilene tape and hook plate fixation were compared. We found that the modified Phemister procedure with CC ligament augmentation resulted in similar functional outcomes to hook plate fixation at the 5-year follow-up point; it also yielded fewer acromial complications, and there is no requirement for a second surgery for implant removal, although the surgical duration was longer, the intraoperative blood loss higher, and the proportion of patients with residual AC joint displacement greater. In light of this comprehensive comparison, the modified Phemister technique with CC ligament augmentation could represent a low-cost alternative surgical option for acute AC dislocation.

Acromioclavicular joint dislocation is a common injury and frequently occurs in active young males. In our series, the average patient age was 43.54 years, with a male dominance (accounting for 65.71%). The optimal treatment option for acute AC joint dislocation remains controversial. Various procedures have been described for the treatment of Rockwood type III to type VI AC joint dislocation [30]. In vertical stability restoration, CC ligament augmentation is one of the mainstays of surgical treatment, with good reported results [15, 16, 31]. Regarding horizontal stability restoration, the hook plate method is appealing, because it provides a rigid fixation and offers the promise of high-stability AC joint fixation, while also maintaining normal biomechanical rotation between the clavicle and scapula [32]. The advantages of this modality are rapid stabilization and reliable reduction maintenance [33]; however, acromial complications and postoperative discomfort are major concerns following the surgery.

In this study, the HK group demonstrated a significantly shorter surgical duration as compared with the PM group. This result was predictable, as during that surgery, we not only established vertical stability, but also horizontal stability of the AC joint, in contrast with other studies [17, 34]. The HK group also presented with less blood loss as compared with the PM group $=delete$. According to a review of the literature, few studies have assessed the intraoperative blood loss in these groups of patients. We postulated that both the higher blood loss and the longer surgical duration in the CC ligament augmentation group resulted from the more extensive anterior deltoid splitting and advanced soft tissue dissection required to allow the curved passer to pass underneath the coracoid process, which is not required in hook plate fixation. However, this low volume of blood loss would not cause major complications in the general population. Regarding neurovascular injury while passing through the coracoid process, no related complications were noted in the present study. However, for patients with multiple trauma or a high anesthesia risk, hook plate fixation might be a better choice due to the shorter durations of anesthesia and surgery.

Cho et al. [13] reported the outcomes of 74 patients treated using the modified Phemister technique with CC ligament augmentation using a suture anchor for acute AC joint dislocation. After an average follow-up of 12.3 months, the authors reported satisfactory clinical and radiological results. Rather than a suture anchor, we used double-strained Mersilene tape for augmentation of torn CC ligaments, which also yielded effective results. Rolf et al. [35] conducted a study of 29 patients managed using a modified Phemister procedure and CC ligament augmentation with polydioxanone (PDS), and also reported satisfactory outcomes after 53 months of follow-up. Our surgical method consisted of the modified Phemister procedure to achieve horizontal stability and CC ligament augmentation for vertical stability of the AC joint. The patients in the PM group had acceptable clinical and radiological outcomes after at least 5 years of follow-up.

Kirschner wires are common implants used to fix the AC joint as an augmentation for temporary stability, and good outcomes have been reported [36]. However, complications such as pin migration or breakage and pin-tract infection are of concern [36, 37]. In our series, only one patient had migration of Kirschner wires, but this had no influence on CCD maintenance. Hook plate fixation provided a significantly greater stability of the AC joint than was observed in the PM group according to CCDR analysis . There were 15 cases of residual subluxation in the PM group (83.3%) and 3 in the HK group, which was statistically significant. Nevertheless, there were no clinical signs or symptoms of instability, and thus no patient required further revision in either group. This indicated that soft-tissue healing of the CC space in the CC ligament augmentation patients could be achieved in a non-anatomic position under the permanent stability provided by a non-resorptive suture material such as Mersilene tape.

Regarding acromial osteolysis, the lateral portion of the hook plate could cause subacromial impingement; in other words, the stress on the base of the acromion increases, which could further result in acromion osteolysis, possibly even complicated by fracture. This poor result usually occurs in patients with a hook plate retention of more than 1 year [24, 38]. This complication could be attributed to an inappropriate size of the implant [23, 38], or interpreted as a force concentration phenomenon due to morphological mismatch between the acromion and hook plate [39]. In our series, there were 4 cases of acromion osteolysis in the hook plate fixation group, although removal of the hook implant was carried out between 4 and 6 months after surgery as previously recommended [23, 24].

## Limitations

There were some limitations to this study. First, the number of cases was limited. Second, the assignment of each group was limited in the retrospective nature. In the comparison of two groups, although there was no difference in preoperative classification, the selection bias might be minimal. But the surgical method depended on the surgeon’s preference, which may be a potential confounding factor. Otherwise, long-term complications such as AC joint arthritis or rotator cuff arthropathy should be further monitored. However, we have nevertheless provided prudent and straightforward results after a minimum 5-year follow-up period in this study.

## Conclusions

Hook plate fixation was superior to the modified Phemister procedure in terms of CC distance maintenance, but there was no significant difference in functional outcome between surgical groups. The modified Phemister procedure with coracoclavicular ligament augmentation using Mersilene tape is a reliable option for acute AC joint dislocation and results in fewer acromial complications than hook plate fixation.

## Data Availability

The datasets used during the current study are available from the corresponding author on reasonable request.

## Abbreviations

AC: acromioclavicular
CC: coracoclavicular
CCDR: coracoclavicular distance ratio
SD: standard deviation
VAS: visual analog score
CMS: Constant-Murley score
UCLA: University of California at Los Angeles
